# Translating weight loss into agency: Men's experiences 5 years after bariatric surgery

**DOI:** 10.3402/qhw.v10.27729

**Published:** 2015-06-09

**Authors:** Eli Natvik, Eva Gjengedal, Christian Moltu, Målfrid Råheim

**Affiliations:** 1 Department of Global Public Health and Primary Care, University of Bergen, Bergen, Norway; 2 Division of Psychiatry, District General Hospital of Førde, Førde, Norway

**Keywords:** Obesity surgery, severe obesity, weight loss, weight loss maintenance, qualitative study, long-term outcomes, lived experience, phenomenology

## Abstract

Fewer men than women with severe obesity undergo bariatric surgery for weight loss, and knowledge about men's situation after surgery, beyond medical status, is lacking. Our aim was to explore men's experiences with life after bariatric surgery from a long-term perspective. We conducted in-depth interviews with 13 men, aged 28–60 years, between 5 and 7 years after surgery. The analysis was inspired by Giorgi's phenomenological method. We found that agency was pivotal for how the men understood themselves and their lives after surgery. Weight loss meant regaining opportunities for living and acting in unrestricted and independent daily lives, yet surgery remained a radical treatment with complex consequences. Turning to surgery had involved conceptualizing their own body size as illness, which the men had resisted doing for years. After surgery, the rapid and major weight loss and the feelings of being exhausted, weak, and helpless were intertwined. The profound intensity of the weight loss process took the men by surprise. Embodying weight loss and change involved an inevitable renegotiating of experiences connected to the large body. Having bariatric surgery was a long-term process that seemed unfinished 5 years after surgery. Restrictions and insecurity connected to health and illness persist, despite successful weight loss and embodied change. Bariatric surgery initiated a complex and long-lasting life-changing process, involving both increased capacity for agency and illness-like experiences.

Severe obesity (body mass index [BMI]>35/40) is more prevalent among women than men, and in Norway one-third of people having severe obesity are men (Midthjell et al., 2013; Ng et al., 2014; Ogden, Carroll, & Flegal, 2014). Having a BMI within or above the range of obesity is associated with decreased life expectancy, impaired quality of life, lower productivity, a range of chronic disorders (type 2 diabetes, coronary heart diseases, stroke, cancer, and osteoarthritis), and increased use of healthcare services (Wang, McPherson, Marsh, Gortmaker, & Brown, 2011; Whitlock et al., 2009). Because severe obesity is understood as a chronic disease in itself, as well as a risk factor, the public Norwegian healthcare services offer an interdisciplinary assessment/evaluation with a possibility of being included in a weight loss intervention, yet not necessarily bariatric surgery (De regionale helseforetakene, 2007; Helsedirektoratet, 2015).

Bariatric surgery^1^ is considered an efficient tool to be used along with lifestyle change to achieve sufficient and sustainable weight loss for people having a BMI within the range of severe obesity (Kushner, 2014; Kushner & Neff, 2013). Bariatric surgery seems to be more efficient in terms of long-term weight loss and reduction of obesity-related comorbidities than other interventions for severe obesity (Adams et al., 2012; Christou et al., 2004; Karlsson, Taft, Ryden, Sjöstrom, & Sullivan, 2007; Pontiroli & Morabito, 2011; Sjöstrom et al., 2004). However, long-term consequences and side effects are scarcely reported in bariatric surgery research (Colquitt, Pickett, Loveman, & Frampton, 2014). Important knowledge might be lacking, and lifelong monitoring and care of bariatric patients are suggested (Buchwald, Ikramuddin, Dorman, Schone, & Dixon, 2011; Madura & DiBaise, 2012; Mechanick et al., 2013).

Typically, about 15–20% of participants in bariatric surgery studies are men (Farinholt, Carr, Chang, & Ali, 2013; Kwok et al., 2014). Against this background, it is assumed that disproportionally few men compared to women are treated with bariatric surgery. As men with severe obesity need the health benefits that are associated with sustainable weight loss just as much as women do, follow-up studies after bariatric surgery highlighting men's outcomes, perspectives, and experiences are important. Men who seek bariatric surgery are generally older and have a higher BMI and more obesity-related comorbidities, compared to women (Arterburn et al., 2009; Farinholt et al., 2013; Livingston et al., 2002; Maciejewski et al., 2012).

Follow-up studies undertaken in US Veteran Affairs Medical Centers are based on a subgroup of predominantly male bariatric surgery patients (73%), and report somewhat different results than the majority of bariatric surgery studies, which include mostly females (Arterburn et al., 2009; Maciejewski et al., 2012). In this group of US veterans, bariatric surgery was not associated with reduced mortality or reduced healthcare expenditures. In one of the studies, male gender and non-Caucasian ethnicity were found to be predictors for not achieving significant weight loss 1 year after surgery, although most participants did (Arterburn et al., 2013).

Few bariatric studies are explicit about gender and gender-related differences, and there are some inconsistencies in the findings that are reported. Some studies have shown that being a male bariatric patient seems related to more complications and adverse events (Livingston et al., 2002; Tiwari et al., 2011). Other studies have not found such differences in complication rates and outcomes after bariatric surgery in men and women, or have shown that men's outcomes are better than women's, in terms of greater weight loss (Andersen, Aadland, Nilsen, & Vage, 2014; Nelbom, Naver, Ladelund, & Hornnes, 2010; Sucandy & Antanavicius, 2013; Tymitz, Kerlakian, Engel, & Bollmer, 2007).

Kolotkin et al. (2008) found that women seeking surgery reported impaired health-related quality of life (HRQOL) and twice the rate of depression compared to men, whereas men reported twice the rate of sleep apnea and higher rates of hypertension and gout than women. They suggested that gender-related differences on HRQOL can be one explanation of why women seek surgery far more often than men. Andersen and colleagues found that female gender, higher BMI at baseline, and nonsmoking predicted lower weight loss 2 years after surgery. Age, employment status, diabetes, and anxiety and/or depression seemed to predict weight loss differently in men and women; increasing age and not having diabetes predicted lower weight loss in men, whereas unemployment and anxiety and/or depression were predictors for lower weight loss in women (Andersen et al., 2014).

Studies based on men's first-person experiences with severe obesity and bariatric surgery are currently lacking. A few studies have investigated men's experiences with overweight or obesity. According to Lewis, Thomas, Hyde, Castle, and Komesaroff (2011), obese men felt responsible for their own body weight and seemed unwilling to seek help. Men understood sedentary lifestyles, stress, lack of work–life balance, and weight-based stigma as causes of weight gain and barriers to weight loss. Sabinsky, Toft, Raben, and Holm (2007) found men's lack of motivation and negative perception of a slimming diet to be barriers to weight loss. The overweight men in their study had desired a lean body and wanted to avoid illness, but their strongest motive for weight loss was to become more efficient at work and a greater asset for their employers.

Taken together, studies based on self-reported data at the group level suggest that there are differences between men and women seeking bariatric surgery and their response to treatment, yet what the concrete differences are and what they mean are not clear. Furthermore, knowledge about male bariatric surgery patients’ situation beyond their medical status is lacking. Participants in qualitative bariatric surgery studies are predominantly females (Bocchieri, Meana, & Fisher, 2002; Engström & Forsberg, 2011; Grønning, Scambler, & Tjora, 2013; Knutsen, Terragni, & Foss, 2013; Ogden, Clementi, & Aylwin, 2006), and some studies explore women's experiences exclusively (Groven, Råheim, & Engelsrud, 2013; Magdaleno, Chaim, Pareja, & Turato, 2011; Warholm, Øien, & Råheim, 2014). As such, new insights into men's bariatric surgery experiences and the meanings attached are needed. In the current study, we explore the research question: What are men's experiences with health and well-being like more than 5 years after bariatric surgery?

## Theoretical approach

We have a phenomenological understanding of the body in illness, health, and well-being (Carel, 2008; Galvin & Todres, 2013; Merleau-Ponty, 1945/2012; Toombs, 1993). The lived body means understanding oneself as an embodied subject and an objective body simultaneously and inseparably, and always connected to the world. Accordingly, bodily being involves a certain ambiguity (Merleau-Ponty, 1945/2012, p. 204). Perception and movement, embodiment and agency, are intertwined. This means that major changes to one's body related to illness might change one's sense of self and one's way of perceiving and inhabiting the lifeworld.

Bodies expressing illness, dependence, passivity, or deterioration might signal vulnerability and diminished longevity, and can be regarded as unwanted, unfortunate, or shameful, whereas bodies expressing fitness, function, and vitality are the most desired (Carel, 2008; Lupton, 2013; Toombs, 1993). Inspired by a phenomenological perspective on bodily being, it is not this study's point of departure to split the meanings of gender into either biological or social ones, or add to that discussion. Our point of departure resonates with De Beauvoir (1949/2000), who acknowledged human life as grounded in human biology, yet claimed that for individuals and society, meanings and values are not determined by biology.

The lived body cannot be reduced to biology *or* subjectivity, nature *or* culture, but can be understood as a *situation*, engaging in all the situations it interacts with (De Beauvoir, 1949/2000, p. 46; Merleau-Ponty, 1945/2012, p. 137; Moi, 2005a, p. 59). The term *situation* points to the body as the fundamental ground for being, and for experiencing oneself and the world. Accordingly, there is no fixed manly or womanly essence to unfold. Rather, it is down to the individuals and the cultures we shape and live in to define the meanings attached to the gendered body throughout life (Moi, 2005b). In other words, our freedom, choice, and responsibility are intertwined with the concrete situation, and the meanings of being a man or a woman vary and are in movement.

## Methods

To explore men's long-term experiences after bariatric surgery, we designed a qualitative study grounded in phenomenology and conducted from a lifeworld research approach (Dahlberg, Nyström, & Dahlberg, 2008b; Giorgi, 2009). The *lifeworld* is the immediate, pre-reflective, and ordinary world in which we live our everyday lives, a meaningful and common world shared through social practices and language (Dahlberg et al., 2008b). Merleau-Ponty (1945/2012) emphasized the lived body as inseparable from the cultural world, and the core from which we perceive, experience, and understand life (pp. 78, 147). The phenomenological lifeworld approach has implications for methodology, analysis, and description of findings in the current study (Dahlberg et al., 2008b; Galvin & Todres, 2013).

We have sought to stay open and sensitive toward the male participants’ experiences and the contexts they are embedded in. We aimed to stay aware of personal beliefs, theories, and assumptions, and to restrain them, because they otherwise could be misleading or limit the research openness toward the phenomenon. The attempt to “bridle” or bracket one's pre-understanding is intertwined with the researcher's open and alert attitude of actively waiting, rather than concluding too early based on one's own preconceptions (Dahlberg, Nyström, & Dahlberg, 2008a). Bracketing does not mean that the researcher can or should forget everything about the phenomenon of investigation, but points to not letting past knowledge decide the present experience (Giorgi, 2009).

### Participants and recruitment

The participants were 13 men who had undergone bariatric surgery between 5 and 7 years before they were included in the study. They were aged between 28 and 60 years, and the median age was 47. Most of them were married or cohabiting and had children, and some lived alone. At the time they were included in the study, most of the men were employed, some were currently unemployed or were on disability leave, and one participant was a student. Some of the men were skilled workers, some had previously been craftsmen but had changed their field of work because of health issues, and a few had tertiary education. They lived in different types of communities, from small rural locations to larger cities in three counties in western and eastern parts of Norway.

All participants had undergone Duodenal Switch, which is a combined surgical procedure that restricts the amount of food possible to eat and limits the nutritional uptake from the food eaten. This bariatric procedure is recommended when substantial weight loss and metabolic control are necessary, but it is associated with higher nutritional risk (Mechanick et al., 2013). Duodenal Switch is used in some clinics because of its effectiveness, especially in patients who have a BMI above or close to 50. The men who participated in the study had lost between 49 and 150 kg after surgery. Most of them had reached a stable weight 5–15 kg above the lowest weight after surgery, some men had been weight cycling and regained 20–30 kg of their weight loss, while a few men had maintained their lowest weight (±5 kg) after surgery.

We sought a variety of rich and vivid descriptions, and asked for men who were interested in participating in an interview and sharing experiences with life after bariatric surgery. In this sense, the group of participants constituted what is often referred to as a *purposive sample* in qualitative research (Dahlberg et al., 2008b; Malterud, 2001; Van Manen, 2014). We recruited participants with the assistance of an experienced nurse at a hospital clinic. The nurse selected men who were able to speak Norwegian and had at least 5 years of experience with life after bariatric surgery. Male bariatric patients who had NN (first author) as a physical therapist during treatment were not invited to participate in the study. We recruited participants in a stepwise manner, meaning that we analyzed portions of data before inviting more participants, until we considered that we had the variation and richness in data needed to gain insight and understanding of the phenomenon (Dahlberg et al., 2008b).

### Interviews

We conducted in-depth research interviews to access the experiential meanings of the men's lifeworld, and encouraged them to describe concrete situations as close to the lived-through events as possible, rather than conceptualizing them (Giorgi, 2009). We developed an interview guide that included an introduction with simple questions about demographical information and the like, a main section covering the broad topics of interest, and some rounding-off questions (Dahlberg et al., 2008b; Giorgi, 2009; Kvale & Brinkmann, 2009).

NN first asked an open question, and followed up by asking about concrete lived-through situations when necessary to obtain rich descriptions. For example, “Could you tell me about your weight loss journey? Can you describe the first situation you remember starting to consider yourself as being too big?” The topics in the interview guide were (1) the weight loss journey, (2) health and well-being, (3) own body, (4) habits related to eating and physical activity, (5) daily life, and (6) relationships. The interviews were undertaken at the participants’ preferred location, and lasted on average 1 h and 20 min. All interviews were recorded and transcribed verbatim by NN (the first author).

### Analyses

Our analyses of the men's experiential descriptions are inspired by Giorgi's phenomenological method (Giorgi, 2009; Giorgi & Giorgi, 2003). In Giorgi's phenomenological method, the movement between the whole empirical material and the parts entails that the researcher first opens up and reads for an impression of the whole, before diving into a thorough analysis of the interviews, one by one, and then finally analyzing across the whole material. Here, we describe this process in further detail. First, the researchers read the interviews for a sense of the whole and tried to see the intentions embedded in the participants’ descriptions.

Second, the researcher went into the interview and divided the empirical description into smaller parts related to the content's meaning. This was done by rereading the interview from the beginning, marking significant shifts of meaning as they appeared throughout the text. In practice, this meant using colors and labeling meaning units with a word related to its meaning and numbers for interview, page, and line. The determination of meaning units was mainly driven by the researcher's spontaneity and lived experience meeting the participant's experience, rather than intellectual resources. This is connected to the phenomenological attitude, and it is one way of being open and sensitive to the empirical data (Dahlberg et al., 2008b).

Third, the researcher went back to the beginning of the description, and transformed and condensed the meaning units from the participants’ natural attitude expressions into descriptions in the researcher's voice. Accordingly, the researcher's descriptions are very close to the participants’, yet are written with sensitivity to the researcher's experiences and perspectives, aiming to clarify the meanings attached. In this process, the researcher sought and tried out various understandings of the meanings by approaching data through asking different questions. To our understanding, this process is what Giorgi (2009, p. 132) has described as free imaginative variation. According to Giorgi, the process of transforming meaning units to condensed descriptions is “the heart of the method.”

Fourth, the researcher wrote a general meaning structure of the men's long-term experience after bariatric surgery based on the transformed meaning units. The researcher aimed to understand and describe the meaning structures of the investigated phenomenon by showing both its invariant meanings and its variations (Dahlberg et al., 2008b; Giorgi & Giorgi, 2003).

### Ethics and consent

The study was approved by the Norwegian Regional Committee for Medical Research. Patients were informed about the study by telephone and received a letter of invitation, including an informed consent form. The participants gave written consent and sent it to NN, who scheduled the interviews. We stated clearly that data would be handled confidentially and that participation was voluntary.

## Results

We present the findings as a phenomenological description (Van Manen, 1997), meaning that we describe the structural features of the men's lived experiences, to show the essential meanings or deeper significance attached to undergoing bariatric surgery. First, we introduce the general structure, identified as “translating weight loss into embodied agency,” which is a condensed description of the findings’ most invariant meanings, and is at the most abstract level of analysis (Dahlberg et al., 2008b). The essential structure includes four constituents, and each constituent is introduced by a short condensed description displaying both the depth and variation in the rich empirical material.

### Translating weight loss into embodied agency

Embodied agency was deeply meaningful and pivotal for the men's experience of a good life. Action and capability to act were existential, meaning that agency and self-understanding converged. Turning to surgery had been a passable way to secure or take back one's own capacity to act, yet weight loss had not spontaneously translated into agency. Navigating tension, thoughts and feelings, altering bodies, and encounters with others was essential for long-term weight loss and change after surgery.

### Becoming a bariatric patient: from large to ill

The men had connected surgery to vanity, which they regarded as valueless and shallow, and did not relate to. They had fought to stay fit for work, maintain family life, and, most importantly, stay independent. Although they had downplayed ill health and disability, the weight-related problems had escalated over time and inevitably demanded attention. The men had fluctuated between being on-guard to avoid the problem of having others’ attention drawn to their bodies and off-guard to approach health services for help. To get access to bariatric surgery, the men had to adopt the perspective that their size was excessive to such an extent that it showed illness. Maintaining autonomy and independence was crucial, but to lose weight for the long term, they felt forced to take action, and their option was bariatric surgery. Consequentially, surgery was eventually considered necessary, life-saving, and thus legitimate, yet imbued with ambivalence from the very beginning.

Most of the men had experienced illness before bariatric surgery. Some had suffered acute illness, such as stroke and myocardial infarction. Others had lived with emotional distress and depression, musculoskeletal pain, chronic cardiovascular diseases, or long-term complications of diabetes. Because they had been ill, their physicians had addressed body weight as a serious health risk and encouraged them to lose weight for years. The men seemed to have neglected that their weight was detrimental for their health, despite the onset of illness and their physicians’ concern. One man said it like this:

I think I needed what that physician said; “If you don't take a new turn, you'll die.”… I really should've realized the severity when I had the stroke. Already back at that time, I should've opened my eyes. It's easy to say [short pause], yet it's not being done [laughing].

In hindsight, he could not really understand how having a stroke in his early 40s had not made him realize that losing weight was crucial to his health. According to the men, this was a common experience, yet most of them had made effort to lose weight on their own, guided by healthcare professionals or following commercial weight loss programs several times. One man said,

Of course you have tried dieting … but every time you tried, you regained the weight plus VAT (Value Added Tax)…. So you had to continue trying everything, right. I have no idea how many times I have tried those things, but it didn't work out. I have tried everything [short pause]. I went to many courses and all, but it wasn't a success. That's why I wanted the surgery so much. It took me 5–6 years before I got it.

Not being able to lose weight had left the men feeling somewhat powerless in the situation. They expressed that feeling powerless had accompanied the emerging realization that they needed help from health care, and considered the physician's introduction to surgery as their chance to accept a new and viable option. According to most of the men, healthcare professionals, friends, or next of kin had initiated their decision about seeking surgical treatment. One man described it like this:

No, I was not really aware that guys did such a thing [having bariatric surgery]…. I don't think of it as cosmetic, because the cosmetic means nothing to me. I know that the people around me think it is all right, no matter how I look. It [surgery] was most for my own well-being…. The doctor opened my eyes, because he said, “If you don't act wisely now, you haven't got that many years left with your grandchildren” [short pause]. Then I thought: My God, I cannot continue like this, it does not work out! We [doctor and man] made a plan and fulfilled it…. I think that it was rather *cowardly* of me to do.

Some men had given up on weight loss attempts because they knew from childhood experience that such efforts had been futile. One man had been very large all his life and had extensive experience of being admonished to eat less and exercise more. He had tried to avoid contact with healthcare professionals and had suppressed his serious weight problems for years. When someone addressed him and his weight differently, he had been taken by surprise, and said,

No one dared to bring up my weight problems before…. Not until some friends talked to a local health care professional, and he came to me and asked me about it. I was at work that day, and then he just came and asked how I was doing. And he started it, talking to my physician and so on. I think that was great [that he came].

Eventually, the men had understood bariatric surgery as their only option and last chance to improve their own health, and they decided that having surgery was the right thing to do. To be introduced to a credible treatment strategy meant being fully recognized as suffering, and it was an essential turning point.

The men described bariatric surgery as an unheroic, inevitable, and necessary strategy to lose weight. They believed that without surgery, they would not be alive at present, or they would live in suffering, confined to disability, illness, and unhappiness. The men had had to re-view and understand the large body as pathological in order to qualify for bariatric surgery. As such, becoming candidates for surgery meant a shift from considering and expressing themselves as large and neglecting health risks, to understanding themselves as seriously ill, risking a shortened life.

### Being “weight lost”: ungraspable transitions

Having bariatric surgery had implied an abrupt and profound change in the men's lives, which affected their entire life. This was described as an unforeseen collapse. The men had lost vital energy and the mental and emotional strength that they had held on to for so long. Most of them had experienced this temporarily after surgery, but for some that was their current situation 5 years after surgery. Weight loss involved radically changed bodies and appearances coupled with profound confusion and feelings of weakness and helplessness. It was a time and situation in which they could not entirely grasp who they were and who they wanted to be.

The radical and profound changes forced them into thoughts and emotions. One of the men described it like this:

What was really difficult related to the bariatric surgery has never been talked about [in health care services]…. Losing weight was a *hard* time…. The physical experience when I lost several kilos per week [pause]; how exhausting and hard it was to lose those incredible amounts of weight. At the same time you had to try to readjust mentally to what happened. One thing is looking forward to being normal weight, but trying to make your mind understand the colossal physical changes in that short amount of time?!… In short, exhausted and confused is how I felt the first two years after surgery…. It is a little ironic and funny, the whole thing, and you just had to laugh at it, really [pause]. One and a half years before, my problem had been tying my shoe laces. Later, I was thin and feeling miserable, and I struggled with functioning in general, because I was so [pause], so exhausted [pause]. I was so exhausted at the time that I didn't reflect on it. I was just in the middle of it, drained and trying to resolve.

Although the surgical change was abrupt, the massive changeover that they needed to accomplish by themselves had lasted for years. They enjoyed losing weight quickly, but being completely exhausted and entangled in a strange and unfamiliar situation had pushed them to their limits. In preparing for surgery, they had stressed surviving the procedure and the physical implications. They had known that surgery required lifestyle change and hoped weight loss would change their lives for the better.

In hindsight, it seemed impossible to prepare for the unexpected drama of the exhausting and vast psychological changes, the emotional intensity, and the mental demands of going through surgery. One man said it like this:

It was *hell* to get back on my feet…. Everything had to do with the psyche…. Being moody, that was the worst…. Often grumpy, I have never been grumpy [before]…. You wanted to be alone. Crabby and grumpy, short-tempered, which irritated me, because that's not who I am. It was periodically like that the first few years…. Eventually, you had to pull yourself together. You went through a lot of phases, and when you realized that you had won the lottery, you just had to find some new solutions…. Then you dealt with it, and tried to stay positive. Because there is nothing to be grumpy about, right. There were no reasons for all that; it's just how it turned out.

The men viewed themselves as self-reliant, yet the confusion and exhaustion after surgery had made them feel helpless in struggling to reorient themselves with their altered bodies in a situation that they had not expected to be in, yet could not escape.

One man who was satisfied with weight loss, his levels of physical activity, and *finding back to himself* expressed concern about being preoccupied with his own body. He had hoped for and expected a slim body and even a *flat stomach*. After surgery, he had become extremely preoccupied with his own body, and he still was. Preoccupation with his own body affected his mind, feelings, and daily life, and he had not told anybody about this struggle before the interview. He said,

Every day, I keep looking at myself in the mirror, just to see if there are any changes, and it has been like that all the time. It [his body] is very much in focus. Before, it didn't matter that much, but now, I am more preoccupied with how I look than I ought to be, a lot more than most other people. I expected to become thin and slim, but I don't see myself as thin and slim, and then I get this focusing [on own body] which I think is no good, because it's like you become obsessed [pause]. It's like you can feel it all the time, feel that you [pause]. I don't know, maybe it turns into a kind of narcotic, that you become obsessed with or *hooked* on it, sort of [pause]. That might be the right word for it, saying that you are hooked on what you look like…. I find it hard to cope with, that I have become totally [sigh], I don't know; hooked on me. It's kind of spooky…. [sighing]. It's [sighing, pause] rather heavy. It's uncomfortable, it's difficult to explain why, but [pause] it's a very unpleasant feeling, to be that preoccupied.

Achieving successful weight loss had not necessarily led to a good relationship with their own bodies; rather, the men expressed that they had been thrown into a devastating confusion. Moreover, they had experienced a split between the overwhelming and always present feeling of being painfully confused and the positive attention and feedback connected to the altering weight loss body. After surgery, the body had demanded an always present awareness from the men, in which some got caught up, and which others made efforts to resist. One of the men who had desperately tried to resist having his own awareness drawn to his altering body said,

It [weight loss] was hell; I mean clothes and such…. It was a barrier…. I didn't feel like it. I can't explain, I thought it was fucking weird. I remember I discussed it with myself and the missus, but I just didn't want to. If I bought those clothes, I thought I wouldn't wear them, because I wasn't ready to wear those clothes [pause]…. I don't think I exaggerate when I say it was one and a half, maybe two years after [surgery]…. Then I bought my first pair of jeans. It was a long process, it sounds weird, I know. Most people say they would run to the store to buy clothes. I was dressed in *huge* sweatpants and procrastinated, I mean, God knows why, to start buying new clothes. Buying clothes in the right size was a problem; you bought them way too large. And I took quite a while to find out which style I liked, what I really wanted…. I remember looking in the mirror, and “That isn't me.” You didn't recognize yourself, it is probably difficult to understand for other people, but you change so enormously and quickly, you have a self-image and that changes…. It was *bizarre*.

The men had intended to leave problems and painful experiences in the past and fully enjoy and embrace the new life. However, the intensity of going through weight loss and change after surgery had forced them into negotiating existential matters, which had caught them by surprise and thus constituted a watershed in their life. The weight loss process had not been uniform and linear; rather, it was chaotic, diverse, and layered. The surgical intervention and its consequences for body, mind, emotion, and social life were coupled with the changes that they had to accomplish and carry out themselves. The desired weight loss was highly appreciated, but nonetheless hard and persistent work to incorporate.

### Revisiting the large body: between social life and embodied shame

Lived experiences and emotional and social burdens connected to the large body resurfaced and became more visible for the men progressively with weight loss, which was described as *revisiting the large body*. Through these revisits, they could renegotiate shame, guilt, humiliation, and failure and develop an expression of greater tolerance for who they had been and still were, regardless of smaller bodies. The men described previously employed strategies in social life, such as always being in a good mood; entertaining, pleasing, or wooing other people; or burying themselves in work. One man said,

I covered a lot with my good mood. I covered a lot because I was playing and singing, and had the focus away from my body…. Yes, I worked *tremendously* with that, I really did. It was probably a lot to hide.

This making up for being too large in parallel with coping with obesity-related problems comprised a double burden. The men had realized that their serious efforts to try to hide and strive to live in the large body had not fully paid off; rather, it had cost far too much energy, and, moreover, the large body had been impossible to hide. After weight loss, seeing a large man or boy in certain situations, such as buying an ice cream on a hot summer day while other people stared and showed their contempt was extremely hurtful and upsetting, and it made the men want to stand up and defend them, or turn away. They were relieved that their bodies no longer attracted negative attention, but they also expressed sadness. It was painful to realize what price they had paid for being large, and the losses some of them had suffered. Most of them had been bullied during childhood and had faced harassment and insults most of their lives because of their body size. One man said,

I have probably changed. I think I used to be more defensive, and it was completely automatic. Now, I feel more confident in myself and others and I'm more relaxed. I got more attention progressively with [quiet laughter] a more normal shape. I experience that people dare to contact me now, more than before. But that was probably because I might have signalled that I didn't want to be in contact with others [pause]. Yes…. From being angry, depressed, and pissed off for 20 years, to now, coping and being open to others…. For me that transformation has been so positive and huge, it's hard for me to find words for it. As I have said, my life started when I was 22 [when having surgery].

This man had withdrawn socially because of his body and expressed a deep gratitude for the weight loss, which had given him courage to connect and interact socially with others. Changing after surgery had both forced and enabled him to deal more deeply with his way of interacting socially. He had discovered that he enjoyed the company of others, and had realized that he was an outgoing and social guy.

The men had different experiences with intimate relationships before surgery. For some, intimate and sexual relationships had worked out; for others, relationships with women had been unthinkable before weight loss. When they had lost weight, some described the joy and excitement of seducing women, having sex, and leading an outgoing life, while others had stayed in, or engaged in new, stable relationships. One man expressed,

Back then when I was a big guy, of course it [having sexual/intimate relations] was not [short pause] somehow an issue for me. I thought that nobody was interested in such a big guy, right…. It wasn't that I didn't [short pause] but I was not that persistent…. I have always loved women, I won't deny that. But one thing is to be in love with a woman; another thing is moving to the stage where you want an intimate relationship and a sex life. Things like that were not really something for me when I was a big guy. I asked for a dance, the girl wanted to dance with me, and I was grateful for that. But to move further [short pause]. There were several sweet girls that were interested [short pause]. It was just inside of me, that I could just forget about that. Yes. Like that [pause]…. It was me, I closed off a bit, I somehow couldn't open up.

In case sexual relationships had come up in conversations when they were large, the men had replied with a quick joke and avoided the subject. Giving words to this situation from the perspective of having lost weight, they underscored that this had been a sensitive and rather serious issue, yet unspeakable for them at that time. None had experienced that physicians had asked them about intimate relationships, sexual life, or the ability to maintain personal hygiene. The men had found it increasingly difficult to carry on their regular hygiene habits as their body weight escalated. This had been a truly sensitive problem, of which they were ashamed, and they had made great effort to hide it.

Becoming helpless and dependent on others had been a worst-case scenario, yet an imminent and inevitable reality they had faced as very large men. Before surgery, they had dreaded the rest of their life as severely obese, ill, and disabled, isolated yet dependent on others. The large and growing body had embodied a threat to the men's entire life; their self-respect, hopes for the future, and for some the will to live. Need for care had implied a full disclosure of embarrassing weight-related problems and giving up on themselves. A few men had lived rather isolated inside their own home and had care from family or healthcare professionals during the last years before surgery.

However, most men had been self-reliant and employed, and some had stayed active socially and physically. Nonetheless, living with severe obesity had been exhausting, and the men had struggled to function sufficiently at work, in the family, and in other social settings. Against this backdrop, the men deeply appreciated all the benefits from surgery. In one man's words,

I can tie my shoelaces, and I can go to the toilet without spilling down my pants. Those things! [Voice breaks]…. Many people don't think about such tiny, tiny little things. I had no chance to scratch my own back. I couldn't reach around myself, and had to lean against a pole. This is not the most important, but still.

Some men were still of a considerable size despite tremendous weight losses, because they had reached a very high weight before surgery. These men particularly voiced a deliberating feeling of freedom, which they connected to no longer being of a sensational size. Weight loss had spared them for having a startling effect on others and made their current life situation a lot easier. One man said it like this:

I'm still large, but not [pause, takes a deep breath]. It's not like you stand out, and everybody just says “*WOW*,” right. You are 1 in 50, and before you might've been one in 1000, if you understand what I mean?

The men described how life had changed, although they still were large; they were able to move and act quite freely in daily life and needed less space, and therefore they could and wished to take part in social life outside of home and work with family and friends. This was a deeply meaningful part of their life, which they labeled as *being active*. As an example, they now had the possibility to travel by plane, although there still were barriers such as small seats, short seatbelts, and other people gazing at them. The men underscored that they felt stronger and less uncomfortable after weight loss in situations when they were being stared at (i.e., others sized them up and checked them out) because their bodies were large.

Despite being large men, they were less self-conscious and uncomfortable after the successful weight loss and the benefits. The men expressed growing self-acceptance of who they were and had been, and the lives they had led. In retrospect, the weight loss and change after surgery had alleviated the shame connected to their own bodies, yet bodily shame was still expressed in relation to weight-related illness, disability, and helplessness.

### The urge for employment: new opportunities, new uncertainties

Employment was an immensely meaningful matter for the men. Living with severe obesity had implied deteriorating health and declining work capability. Because weight-related ill health nearly had pushed them out of employment, weight loss maintenance and health currently were significant issues. Weight loss had made work tasks more possible to undertake, and employment was easier because it no longer drove the men to exhaustion.

After weight loss, the physical and social barriers connected to their size had vanished, and the employed men expressed confidence, mastery, and competence at work.

I have the same job that I used to, it's a quite physical job; I drive a large vehicle, involving climbing, carrying and so on. I have often thought that it's [knocking the table] a wonder I didn't have a heart attack or something, because carrying the weight that I did [sighs]…. I used to be completely *crushed* before. To fetch a container was such a physical strain, that I compare it with having had a hard workout…. Sometimes, when you climbed to the top to secure the container, it was such a heavy load, and you climbed back down and it was as if you couldn't get enough air; you had to calm down, you were shaking, feeling the pressure in your head. But now, I don't mind, I enjoy it…. My boss never said anything, but he has commented on it lately; although I seem calmer and easier, I produce about twice what I did before.

One of the skilled workers described that not only his capacity but also his way of organizing and performing his professional life had radically changed. His unique qualifications and skills had helped him to stay employed, but it had become impossible for him to practice his profession with his own hands, because of severe weight problems. He said,

These young lads did not know how to do it, right. But I could explain to them what to do, and they did what I told them to. Because I could not go down there myself, I couldn't get at it…. There were places I could not get in, right…. But now, I do everything. I think it is fun, because I get in everywhere, I get to everything now! I mean [short pause] it's a *terrible* restructuring, and a massive change in the way of working.

The same man still supervised and worked with groups of young apprentices, but expressed joy and freedom of choice; he could do everything by himself if he wanted to. The men on disability leave, the students, and the unemployed believed that weight loss had increased their chances to become employed, which they all wished for. One man who had been on disability leave for years said, “I would like to do something more, to be useful. That feeling, it's there. *That's* why I want to try returning to the labour market.”

Some of the men suffered from complications or severe side effects, or had experienced onset of disease after surgery. Despite weight loss and improved health in some ways, they currently lived with chronic illness, and thus being employed had become a demanding and distressing situation in new ways. One man said,

It's my skeleton [pause]…. Pain in the knees, pain in the hips, it's all over, in the back and so on. You think “Oh my God, how can I go to work?”, but you take a shower, standing there for half an hour, and then you are fit to fight. It was never like this before…. I can feel my bones bruise easily. Rib fractures and the like [short laughter and pause] have happened to me many times. I can't do what I used to. When you have a container in the garden and just bend over to throw something into it; I can't do that anymore, because then they [the ribs] break or bruise. I just got confirmed that it is [pause] osteoporosis…. You know, when you just lean over a chair, and feel yourself bruise or break, it's not a good feeling…. I can't go running anymore, but I can still walk which I also like a lot, so that's not a problem…. I'm sceptical about the future, because I struggle with my skeleton and my body. That's the heaviest burden [pause]. I really like working, and I fear that I might have to stop because I just can't do it. It's kind of scary. The biggest hindrance for me to the future is if I have more problems with my body and my skeleton.

The risk of again falling out of employment or needing disability leave because of ill health was an unsatisfactory and uncertain situation to be in after bariatric surgery. Some of the men lived in families in which the spouse suffered from chronic illness and one or more of the children were chronically ill or disabled. Their families needed them to be healthy and functional, to support the family financially as caregivers, and to be fit for solving practical issues at home. The men expressed that ill health and giving up employment were not options in their current life situation. They struggled and kept pushing themselves at work and at home, suppressed signs and symptoms of illness, and held up the hope that their health would improve or change for the better.

The men had approached bariatric surgery hoping to become capable of doing more of what added value to their lives, and having a job and working appeared as crucially important. Their approval of work was connected to controlling their finances and social situation, exercising self-discipline, recognizing self-worth and self-respect, and being responsible and caring for their families. Despite successful weight loss, the men expressed insecurity about whether the body and health could be trusted for the future, given the types and severity of illness some of them lived with. Ill health that interfered with their capability to act freely and functionally in life carried a profound threat to who they were and wanted to be, and the future ahead for themselves and their families.

## Discussion

The freedom of being, choosing, and acting without weight-related limits was a profound relief creating significant and ongoing changes in the men's lives. The men in the current study experienced active engagement with the world as deeply meaningful and expressed themselves as *doers*, meaning that their self-understanding seemed inseparable from their actions. Within phenomenology, *agency* means inhabiting and exercising a possible freedom to act within certain limits, make choices, and be held accountable for one's actions (Galvin & Todres, 2013, p. 12). The lived body is our power for action and for having a world (Merleau-Ponty, 1945/2012, p. 111). Actions begin with perception of the environment and spontaneous embodied engagements connected to the task at hand, and thus go beyond mere causal responses to physiological stimuli (Romdenh-Romluc, 2011, 2013). Thus, severe obesity had constituted a threat not only to the participants’ health and longevity, but also to their existential being.

From the perspective of existential phenomenology, we are born as free subjects and defined by our actions; we are what we do (De Beauvoir, 1949/2000; Moi, 2005a, p. 55; Sartre, 1943/2003). To be meaningful, subjective freedom needs to be actively and consciously taken up and lived, and it includes openness for others’ freedom. Thus, freedom involves choice, agency, uncertainty, and responsibility. Because freedom cannot be separated from our situation, Beauvoir made a distinction between existential and *concrete* freedom; social, political, or material living conditions, including various forms of oppression, can restrict our concrete freedom, choice, and bodily being (Moi, 2005b, p. 229). Although Beauvoir concentrated on women's situations, within the present context her understanding of freedom seems relevant for both women and men.

The men experienced a tension between *holding on to* what they regarded as meaningful in their lives and changing their bodies, habits, and practices for the long term. Accordingly, they made an effort to hold on to some of their own ideas, initiatives, projects, and plans. The men expressed this as intertwined with life prior to surgery, concentrating on staying confident in own abilities and capabilities, despite the weight-related threat to their health, well-being, and practical and social lives. This continuity was deeply meaningful and seemed to strengthen the basis for accomplishing changes after surgery. The intimate relation between time and subjectivity can shed light on the participants’ emphasis on continuity. Past experiences intertwine with our present being and reach toward the future (Merleau-Ponty, 1945/2012, p. 332). As such, we are not *either* captured in our past *or* able to just move on. The men in our study expressed that being self-reliant had supported them in both managing a challenging life as large men and living through a multitude of changes after surgery. As such, we understand self-reliance as a potent and valuable resource for men's health and well-being, and not only a barrier to seeking help from health care.

Although the weight had threatened the participants’ lives, they habitually had conceptualized weight-related problems as personal issues that they had to handle or cope with on their own. The men expressed a tension between being self-reliant and being a patient, and expressed initial resistance to strict normative perspectives on body size, health, habits, and practices, which they did not identify with. However, at the point when the surgery became an option and their weight-related problems had become unmanageable, they were ready to expand the experiential perspective to include the health/medical perspectives on obesity. Accordingly, they accepted large body size as obesity: a major risk for cardiovascular diseases, diabetes, physical disability, and psychological distress, and a threat to public health (World Health Organization, 2014). As such, from the start, bariatric surgery meant *taking action* and not giving in.

The men wanted to be active agents in their weight loss processes, yet most of them had experienced a situation of absolute exhaustion, and had been less capable during recovery and weight loss than before surgery. Unprepared for the profound exhaustion and emotional demands, the men experienced a deep insecurity about the capability to live the lives they found meaningful and had longed for. Accordingly, the first years of postsurgery life were described as much like living with illness, even though they recovered from weight-related illness and psychological distress.

*Illness* is described as a disruption of the lived body, which means becoming aware of one's own body as a body, rather than living with it unreflectively (Toombs, 1993, p. 70). With parallel to Toombs’ description of living with illness, bariatric surgery made habitual engagement with the world difficult and involved worrying about new threats to the men's future projects. The men expressed feeling thrown into a new and unfamiliar situation; they were forced to embody a variety of postsurgical changes, yet felt somewhat deprived of the habitual power to act, and without the possibility of escaping. They all expressed this connected to rapid weight loss and embodied change, and highlighted the first 2 years as the most intense phase in their process of change. For some, such threats had resurfaced with late complications.

These findings have parallels to studies showing that the first years after gastric bypass surgery are infused with ambivalence related to surgical weight loss and lifestyle change for female bariatric patients (Groven, Råheim, Braithwaite, & Engelsrud, 2013; Groven, Råheim, & Engelsrud, 2010: Groven, Råheim, & Engelsrud, 2013; Warholm et al., 2014). In illness, otherness points to negative and profoundly alienating experiences (Carel, 2008; Toombs, 1993). After bariatric surgery, otherness points to weight loss and change and is understood as positive. In the light of our findings, change is far more complex and does not follow a linear and univocal trajectory. Major weight loss made the men's bodies less different when compared to other bodies, yet radically different when compared to their habitual bodies, for the better and for the worse.

The participants’ intense emotional experiences related to weight loss and embodied change were profoundly distressing, difficult to go through on their own, and confusing to such an extent that they still were wondering and thinking about it. The confusion seemed connected to not understanding themselves, nor being understood as ill, yet living through an illness-like experience. The intensity expresses the existential depth of the men's experiences. In Toombs’ (1993) words, “illness necessarily incorporates not only a threat to the body, but a threat to one's very self” (p. 70). The obesity literature often refers to high levels of obesity as a chronic illness. According to our findings, the habitual large body was not necessarily experienced as illness, yet the postsurgery body was.

According to Groven's dissertation (2014), female bariatric patients had experienced severe obesity as a deeply unacceptable situation, both socially and personally. To satisfy the social demands for a healthy and feminine body, Groven questioned whether the women had a choice or rather were driven into bariatric surgery. Our findings show that the men had suffered under the stigma related to obesity, too, yet their main concern was disability. It is important to note here that the men in our study had been very heavy and had suffered from obesity-related illness, which is typical for the male bariatric population. The female bariatric patients strove for healthy lives (Groven, 2014), whereas the men in the current study wanted surgery to secure and increase their capacity to act, and avoid disability and dependency.

The men in the current study conceptualized bariatric surgery as taking *responsibility* for their own health and the others whom they cared for, and who were dependent on them. In research on men and health, Robertson and Williams identified a dilemma for laymen in taking care of their own health; the “don't care/should care” dichotomy. The dilemma points to not being too concerned about health because it is understood as a feminine issue, or aiming to be “good citizens” and therefore having a moral obligation to engage in healthy practices (Robertson & Williams, 2010, p. 50). The men in our study seemed not to identify with the idea that striving for weight loss was womanish, or that surrendering to surgery might signal loss of control or lack of power to change their own lives. Rather, they expressed a moral obligation to do whatever it would take to lose weight and gain or improve their own health.

Bariatric surgery is described as a *tool* for lifestyle change by healthcare professionals and bariatric patients (Groven, Råheim, Braithwaite, et al., 2013). The tool metaphor points to surgery as a facilitator for losing weight, underscoring that the responsibility to continue healthy eating and keep up sufficient levels of exercise are in the patients’ hands. Accordingly, the patients have the power to produce the desired outcomes after surgery through an altered lifestyle. These are the same patients who have tried and not succeeded to accomplish lifestyle change, and therefore have surgery.

Accordingly, patients face high expectations; they should adapt to the changes and consequences of surgery, including side effects and complications; take part in the follow-up services; and put the advice they get into practice. Being responsible and active in changing one's life while living through a new and illness-like experience creates a situation under pressure, as described by the participants in the current study. Bariatric surgery might be understood not only as a tool but also as a gift from society to carefully selected persons. The expectations to bariatric patients might bear the undertone of a return favor, which is taking responsibility for one's own health by living healthily and keeping the weight off. If they somehow fail, they run the risk of again facing stigma related to obesity, experiencing double shame for being unsuccessful after surgery, and feeling blame and self-blame for their own situation (Groven, Råheim, Braithwaite, et al., 2013). Thus, expecting a nearly linear process from surgery to sustainable weight loss, accomplished through patient-driven change, might be harmful.

Although losing weight was life-saving and deeply meaningful to the participants, there was a gap between surgery, follow-up, and their experienced need for care and support. Self-care and efforts to live a healthy life were issues addressed in follow-up, whereas the men's search for meaning and identity was not. Their sense of self and self-reliance had been deeply challenged after bariatric surgery. We understand these findings in light of Fuchs (2005) claim that reductionist approaches to treatment might undermine patients’ self-understanding, self-efficacy, and autonomy. Bariatric surgery is effective, yet it remains a localized and narrow approach to a complex problem. After all, severe obesity is connected to environmental aspects like the culture of excess, the vast availability of energy-dense foods and drinks at relatively low cost, and no need in particular to use the body to accomplish daily living, and not only to individual aspects like genetics, choices, or lifestyles (Swinburn et al., 2011).

According to phenomenology, person and world are intertwined, and past experiences, habits, and the cultural context are not simply left behind (Landes, 2013, p. 102). The dominating emphasis on weight loss and change after bariatric surgery is understandable, but in light of our findings and those of other qualitative studies, it remains skin-deep. Our findings show that men who had undergone bariatric surgery strongly identified with their actions, and having the possibility to make choices was deeply significant. We understand these men's surgical weight loss projects as a quest for concrete freedom and meaningful lives. Translating weight loss and embodied change into agency was pivotal for life after bariatric surgery, a situation infused with ambiguities for the long term.

### Methodological considerations

In phenomenological studies, an open and sensitive attitude is imperative (Dahlberg et al., 2008b; Giorgi, 2009; Nordlyk & Hardner, 2010). We have spelled out how we understand and apply core methodological ideas within phenomenology, and shown what openness means in the current study. We hope that the reflective scrutiny concerning our own pre-understandings and openness toward the participants’ lived experiences comes through in the current article. The men's rich experiential descriptions created wonder and gave good possibilities for analysis and insight (Van Manen, 2014).

We have offered a meaning structure of men's experiences after bariatric surgery, which gives possibility for deeper reflection and new insight (Van Manen, 1997, 2014). In displaying essential features of the phenomenon, including its variations, we hold the findings to be strong. We underscore that the meaning of a particular phenomenon cannot be fully described once and for all. Understanding and meaning are infinite; meaning is connected to the lifeworld, which might change, and then meaning changes too (Dahlberg et al., 2008b, p. 115).

Being transparent about the research process and explicit in presenting findings, we believe it is possible for the readers to make their own opinion about to which extent, in which ways, and to what other situations the presented findings could be of value (Dahlberg et al., 2008b). Van Manen (2014) suggested that phenomenologists consider *existential generalization*, which is the possibility to recognize aspects of meaning attached to a particular phenomenon, and *singular generalization*, which points to the possible uniqueness of a description (p. 352). Accordingly, the generalizability of the current findings must be considered carefully, and with respect to the certain type of insights and knowledge phenomenology can offer.

### Implication for practice

In light of the findings presented, men might need to be invited to dialog about the meanings at play in the inevitable embodied and existential process of change after bariatric surgery. Inspired by an approach for humanizing care, outlined by Galvin and Todres (2013), we propose a health care that assists and facilitates the process of making patients responsible for their bodies and supporting them in regaining control of their health. A humanizing approach seems particularly relevant in this field. Interventions like surgery, blood samples, dietary supplements, and the like express limited time and less openness for dialog, wondering, or emphasis on exploring the profound, life-changing, and ongoing process.

## Note

1 The term *bariatric surgery* points to different surgical procedures used to facilitate weight loss via restriction of the capacity for nutritional intake, or restriction combined with malabsorption of nutrition (limited nutritional uptake from the digestive tract). Worldwide, adjustable gastric banding is the most-used restrictive surgical procedure, whereas gastric bypass is the combined procedure that is most frequently performed.
